# Volatile organic compound profiling by HS-GC-IMS for vaginal infection identification

**DOI:** 10.3389/fcimb.2026.1827164

**Published:** 2026-05-05

**Authors:** Peng Liu, Haiyan Zhou, Rongguo Li, Xiaodi Chen

**Affiliations:** Clinical Laboratory Department, Jinan Maternity and Child Care Hospital Affiliated to Shandong First Medical University, Jinan, China

**Keywords:** bacterial vaginosis, biomarker, HS-GC-IMS, volatile organic compounds, vulvovaginal candidiasis

## Abstract

**Background:**

Bacterial vaginosis (BV) and vulvovaginal candidiasis (VVC) are common vaginal infections; however, the existing techniques for diagnosing these infections are often subjective and inefficient. This study examined the ability of headspace-gas chromatography-ion mobility spectrometry (HS-GC-IMS) to diagnose BV and VVC by analyzing volatile organic compounds (VOCs) in vaginal swabs.

**Methods:**

A total of 616 study participants were recruited, comprising 152 patients with BV, 174 patients with VVC, and 290 healthy controls. Participants were randomly allocated into a training cohort (n = 432) and an independent test cohort (n = 184). Vaginal VOC profiles were examined using HS-GC-IMS. A partial least squares discriminant analysis (PLS-DA) model was developed to differentiate the groups and identify unique biomarkers for BV and VVC.

**Results:**

Fifty-nine VOC peaks were identified among the samples. The PLS-DA model exhibited strong classification efficacy, with overall predictive accuracies of 84.26% on the training cohort and 80.98% on the test cohort. A receiver operating characteristic analysis produced area under the curve values surpassing 0.90 for all groups, signifying high model reliability. Eight distinct VOCs were identified as potential diagnostic biomarkers. Based on these biomarkers, the PLS-DA model had an overall prediction accuracy of 78.25% across the entire cohort of 616 participants.

**Conclusion:**

HS-GC-IMS provides a rapid, sensitive, and non-invasive method for characterizing vaginal VOCs. The constructed model accurately distinguished BV and VVC, indicating that this technique has considerable potential as an innovative clinical instrument for objectively identifying vaginal infections.

## 1 Introduction

As an integral component of the human microbiome, the vaginal microbiome is essential for sustaining female reproductive health (Liu et al., 2023b; Brennan et al., 2024). In healthy women of reproductive age, the vaginal microbiome predominantly comprises *Lactobacillus* species, which maintain a balanced ecology (France et al., 2022; Landolt et al., 2025). However, when the vaginal microbiome equilibrium is disturbed, vaginal dysbiosis may result, which may lead to various infections. Bacterial vaginosis (BV) and vulvovaginal candidiasis (VVC) are the two predominant forms of vaginal infections encountered in clinical settings. There is a significant global incidence of these two conditions, with roughly 23–29% of women of reproductive age experiencing BV and 75% of women experiencing VVC at least once in their lifetime; however, the current state of clinical diagnosis of these infections is concerning (Chen et al., 2021; MacAlpine and Lionakis, 2024). Conventional diagnostic techniques predominantly use microscopic examinations, which are limited by considerable subjectivity, a slow turnaround, and high labor requirements (Lokken et al., 2022; Amerson-Brown, 2025).

In recent years, clinical metabolomics and volatilomics have emerged as novel means of diagnosing infectious diseases (Capuano et al., 2025; Dell’Olio et al., 2025). Every microbial species has a distinct array of unique metabolic pathways that are precisely encoded by its DNA and honed during extensive evolution. Bacteria and fungi produce distinct profiles of microbial volatile organic compounds (VOCs) into the extracellular environment during processes such as growth, colonization, nutrition acquisition, fermentation, and quorum sensing-mediated communication (Navarro-Laguna et al., 2025). The distinctive emissions collectively form a highly precise and pathogen-specific ‘odor fingerprint’, providing critical information related to the pathogen type and metabolic conditions (Drees et al., 2019; Salinas-García et al., 2025).

Headspace-gas chromatography-ion mobility spectrometry (HS-GC-IMS) is an innovative analytical method that combines the superior separation capabilities of gas chromatography with the high sensitivity and rapid detection of ion mobility spectrometry at atmospheric pressure, with significant potential for detecting trace VOCs in complex biological samples without complicated sample preparation (Gao et al., 2024; Lan et al., 2025; Zhao et al., 2025). The clinical value of HS-GC-IMS has been extensively validated across multiple fields as a powerful, non-invasive diagnostic tool. Recent applications in physiological screening have demonstrated its potential in identifying human-derived volatile biomarkers via sweat analysis (Tungkijanansin et al., 2024). The diagnostic capabilities of GC-IMS have extended beyond sweat to encompass the profiling of serum VOCs, presenting promising non-invasive methods for the early identification of malignancies, including gastric cancer and hepatocellular carcinoma (Zhao et al., 2026; Shen et al., 2025). More importantly for clinical microbiology, HS-GC-IMS is proving to be a transformative tool for rapid pathogen differentiation. Previous research has shown that it can effectively analyze intricate volatile profiles to detect distinct microbial VOCs in mixed bacterial growth conditions (Lu et al., 2022). Recent research underscores its effectiveness in profiling urine VOCs, which, when integrated with machine learning algorithms, may swiftly diagnose urinary tract infections and precisely identify the underlying bacteria (Zheng et al., 2026). In demanding and time-critical clinical settings, this method is utilized in the intensive care unit through breathomics to swiftly detect the microorganisms responsible for ventilator-associated pneumonia in minutes (Schnabel et al., 2015). Furthermore, HS-GC-IMS facilitates continuous, non-invasive surveillance of blood culture bottle headspace for the early detection of sepsis, issuing alerts for the proliferation of life-threatening bacteria hours or even a full day before to traditional colorimetric carbon dioxide monitoring systems (Drees et al., 2019).

In this study, we used HS-GC-IMS to identify BV and VVC by analyzing the VOC profiles of vaginal swabs. This method provides novel insights into the rapid identification of vaginal infections, with significant potential for clinical application.

## 2 Materials and methods

### 2.1 Chemicals and materials

2-Butanone, 2-pentanone, 2-hexanone, 2-heptanone, 2-octanone, and 2-nonanone (analytical reagents, 99.999%) were acquired from Aladdin Biochemical Technology Co., Ltd. (Shanghai, China) as reference standards. High-purity nitrogen (≥ 99.999%) and 20-mL headspace vials were supplied by Jinan Deyang Special Gas Co., Ltd. (Jinan, China) and Shandong Hanon Scientific Instruments Co., Ltd. (Jinan, China), respectively. Swabs for collecting vaginal secretions were obtained from Jiangsu Kangjian Medical Apparatus Co., Ltd. (Taizhou, China).

### 2.2 Sample collection and vaginal microecology testing

Study participants were recruited between 2023 and 2024 in the Jinan Maternity and Child Care Hospital Affiliated with Shandong First Medical University. The inclusion criteria were as follows: (1) Patients refrained from vaginal douching and intravaginal medicine for 3 days before sample collection. (2) Patients abstained from sexual intercourse for 24 hours prior to sample collection. (3) Samples were not obtained during the menstrual cycle.

A sterile, dry cotton swab was used to collect a sufficient volume of discharge from the posterior fornix of the vagina of each participant. Each specimen was then transferred to a disposable plastic vial. A drop of the vaginal sample suspension was placed at the center of a slide, dried, fixed, and Gram-stained. Samples were evaluated for the presence of vaginal epithelial cells and bacterial flora under a low-power microscope and an oil immersion microscope. The results were analyzed using the Nugent scoring system to diagnose BV. The composite score was classified into three categories: scores of 0–3 indicate health, 4–6 signify an intermediate status, and 7–10 show definitive BV (Nugent et al., 1991). To diagnose VVC, each sample was then prepared as a suspension by adding six to eight drops of a diluent to the sample on a glass slide. A wet mount was then prepared and observed under a low-power microscope and a high-power microscope to detect fungal hyphae and spores (Sobel, 2007). Vaginal swabs with negative findings in tests were classified as healthy.

### 2.3 VOC analysis using HS-GC-IMS

A GC-IMS system (FlavourSpec^®^, G.A.S., Dortmund, Germany) was equipped with a CTC PAL autosampler (CTC, Zwingen, Switzerland).

To prepare the HS samples, the vaginal cotton swab heads were removed and promptly placed in headspace vials and sealed. The samples were incubated at 80 °C for 15 min with an agitation speed of 500 rpm. The temperature was intentionally chosen above physiological settings (37 °C) to supply adequate thermal energy to surpass matrix retention effect in order to optimize the volatilization and headspace enrichment of heavier semi-volatile microbial biomarkers. A 15-minute incubation at 80 °C is optimal for releasing stable metabolites while remaining far below the thermal thresholds that generate substantial artifactual VOC formation. Then, 1000 µL of the headspace sample was injected in splitless mode, with the syringe temperature held at 110 °C.

Gas chromatographic separation was performed using an MXT-WAX capillary column (15 m × 0.53 mm ID, 1.0 µm film thickness; Restek, Bellefonte, PA, USA). The column temperature was maintained at 60 °C. The carrier gas was high-purity nitrogen. The flow rate was as follows: 2.0 mL/min for 2 min, gradually increased to 50.0 mL/min over 8 min, and then increased to 100.0 mL/min over 5 min. The overall duration was 15 min, and the inlet temperature was 80 °C.

IMS was performed using an IMS detector outfitted with a tritium ionization source. A drift tube (length, 98 mm) was used at 45 °C with a constant electric field intensity of 500 V/cm. The drift gas was high-purity nitrogen at a flow rate of 150.0 mL/min. The analysis was conducted in positive ion mode. The gate open duration was 100 µs, and the closure voltage was 80 dgt.

VOCs were determined by analyzing the GC-IMS data using VOCal software (version 0.4.35; G.A.S., Dortmund, Germany), including the integrated Reporter and Gallery Plot plugins to visualize three-dimensional, two-dimensional, and fingerprint chromatograms of the VOCs. C4–C9 n-ketones were used as a benchmark to calculate the retention index (RI). Qualitative identification of VOCs was performed by matching the obtained RI against the National Institute of Standards and Technology 2020 RI database and the relative drift times (Dt) against the Hanon 2025 Dt database. During data analysis, the raw Dt was normalized to the Reactant Ion Peak to compute the relative Dt, thus reducing micro-fluctuations in the instrument. Strict two-dimensional tolerance limitations were enforced for accurate identification: a RI deviation of ≤ ± 30 and a relative Dt deviation of ≤ ± 0.02. Any VOC peak over either of these limits was unequivocally categorized as unidentified.

### 2.4 Quality control and blank analysis

To confirm that the identified VOCs only emanated from the clinical samples and to mitigate potential background contamination, stringent blank analyses were systematically performed during the investigation. Three categories of blank samples were concurrently evaluated with the clinical cohorts: environmental air blanks, instrument blanks (empty sealed vials), and swab blanks (sterile, unused vaginal swabs placed in headspace vials and incubated at 80 °C for 15 minutes). During data processing, any background VOC peaks from the swab materials, plastic vials, or laboratory environment were rigorously eliminated.

### 2.5 Statistical analysis

Statistical analyses were conducted on the peak heights of all data using SIMCA software (version 14.1; Umetrics, Umea, Sweden). A partial least squares discriminant analysis (PLS-DA) model was developed to identify possible biomarkers based on variable importance in projection (VIP) scores. Potential biomarkers were selected based on the following criteria: VIP > 1 and *P* < 0.05.

## 3 Results

### 3.1 Clinical characteristics

This study included a cohort of 616 individuals with verified diagnoses: 152 patients with BV, 174 patients with VVC, and 290 healthy women. By using SIMCA software (version 14.1), 70% of the enrolled participants (n = 432) were randomly assigned to the training cohort (n = 106 BV, n = 127 VVC, and n = 199 healthy), while the remaining 184 participants (n = 46 BV, n = 47 VVC, and n = 91 healthy) comprised the test cohort. There were no significant differences in age across the three groups (*P* > 0.05).

### 3.2 VOC profile analysis by HS-GC-IMS

**Figure 1:**
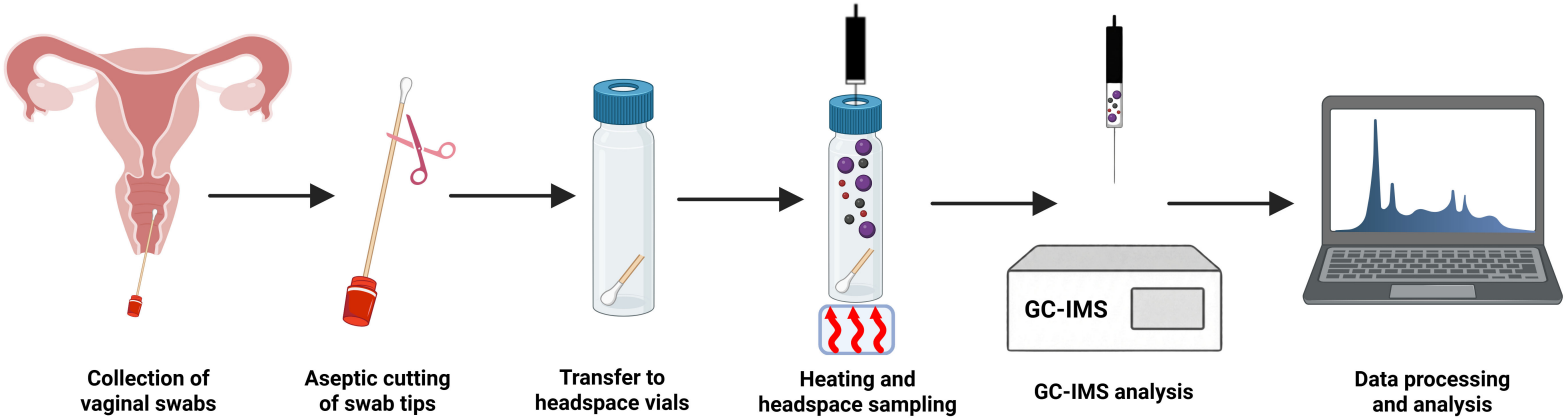
Procedure for identifying VOCs in the vaginal swabs. The procedure included the acquisition of vaginal swabs. The swab tips were removed using sterile technique, and samples were placed into headspace vials for thermal incubation and headspace extraction. The resulting VOCs were examined using GC-IMS, followed by data acquisition and a chemometric evaluation.

**Figure 2:**
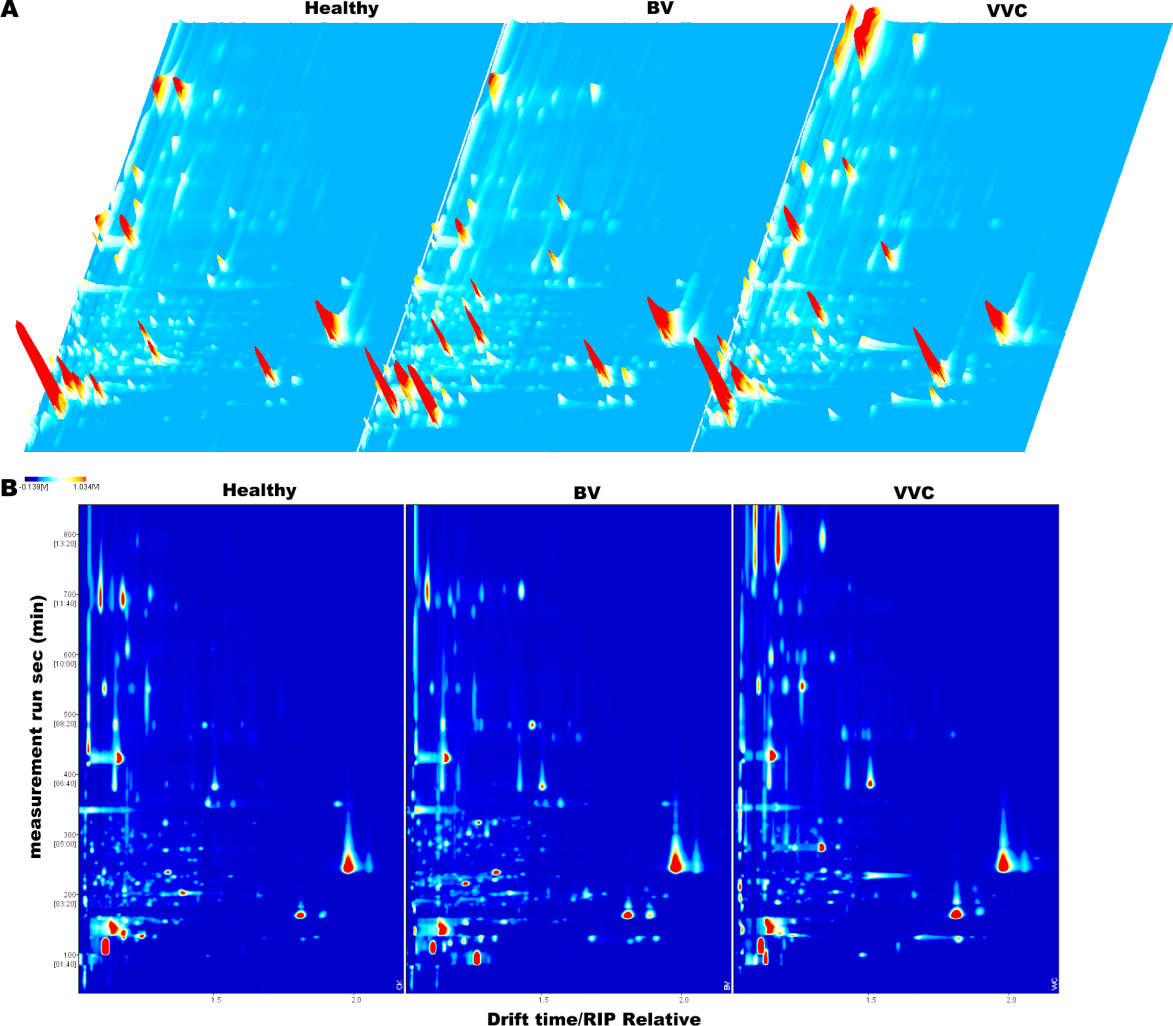
HS**-**GC-IMS spectra of VOCs in the vaginal swabs of different groups. **(A)** Three-dimensional spectrum; **(B)** two-dimensional spectrum.

We developed a standardized analytical process to systematically identify VOCs from vaginal samples, as depicted in **Figure 1**. Vaginal swabs were collected and underwent sterile preparation, in which the swab tips were removed and placed into headspace vials. After thermal incubation and headspace extraction, the samples were analyzed by GC-IMS. The analytical results of each sample were visualized as three-dimensional (3D) and two-dimensional (2D) topographic spectra. As shown in **Figure 2A**, VOCs were successfully separated in the 3D visualization, but the research groups could not be successfully distinguished visually due to their spectral similarities. Thus, 2D topographic spectra (**Figure 2B**) were used to evaluate quantitative variances. The signal intensities of the 2D spectra were color-coded (red: high; blue: low) to enhance the comparative analysis across samples. Using VOCal software (version 0.4.35) with a GC-IMS library, a total of 59 VOC peaks were manually selected from the samples based on their RI and Dt. The peaks included 47 identified substances and 12 unknown substances (**Table 1**).

**Table 1:** VOCs detected in the vaginal swabs.

No.	VOC	CAS #	Formula	MW	RI	Rt (s)	Dt (a.u.)
1	Acetic acid-M	64-19-7	C_2_H_4_O_2_	60.1	1447	430.357	1.058
2	Acetic acid-D	64-19-7	C_2_H_4_O_2_	60.1	1445	428.698	1.165
3	1-Nonanal-M	124-19-6	C_9_H_18_O	142.2	1355	353.513	1.482
4	1-Nonanal-D	124-19-6	C_9_H_18_O	142.2	1353	351.835	1.944
5	Trimethylamine	75-50-3	C_3_H_9_N	59.1	641	76.502	0.964
6	2-Propanone	67-64-1	C_3_H_6_O	58.1	811	110.034	1.117
7	Tetrahydrofuran	109-99-9	C_4_H_8_O	72.1	866	124.006	1.228
8	2-Butanone	78-93-3	C_4_H_8_O	72.1	899	132.948	1.252
9	2-Pentanone	107-87-9	C_5_H_10_O	86.1	970	154.744	1.369
10	Toluene	108-88-3	C_7_H_8_	92.1	1038	179.054	1.012
11	Acetic acid ethyl ester	141-78-6	C_4_H_8_O_2_	88.1	882	128.197	1.338
12	Cyclopentanone	120-92-3	C_5_H_8_O	84.1	1174	239.611	1.340
13	2-Methyl-1-propanol-M	78-83-1	C_4_H_10_O	74.1	1096	202.935	1.184
14	2-Methyl-1-propanol-D	78-83-1	C_4_H_10_O	74.1	1097	203.145	1.387
15	Ethenylbenzene	100-42-5	C_8_H_8_	104.2	1210	259.101	1.415
16	2-Butanone, 3-hydroxy-D	513-86-0	C_4_H_8_O_2_	88.1	1240	276.461	1.334
17	2-Butanone, 3-hydroxy-M	513-86-0	C_4_H_8_O_2_	88.1	1238	275.242	1.070
18	1-Hydroxy-2-propanone	116-09-6	C_3_H_6_O_2_	74.1	1257	286.455	1.066
19	1,3-Diaminopropane	109-76-2	C_3_H_10_N_2_	74.1	1340	342.031	1.063
20	1-Hexanol-M	111-27-3	C_6_H_14_O	102.2	1308	319.162	1.331
21	1-Hexanol-D	111-27-3	C_6_H_14_O	102.2	1311	321.291	1.648
22	Benzaldehyde	100-52-7	C_7_H_6_O	106.1	1501	483.347	1.471
23	Propanoic acid-M	79-09-4	C_3_H_6_O_2_	74.1	1557	544.842	1.112
24	Propanoic acid-D	79-09-4	C_3_H_6_O_2_	74.1	1558	545.489	1.269
25	Methyl 3-methylthiopropionate	13532-18-8	C_5_H_10_O_2_S	134.2	1502	484.641	1.602
26	2-Methyl-propanoic acid	79-31-2	C_4_H_8_O_2_	88.1	1595	591.094	1.157
27	2,5-Dimethyl-4-methoxy-3[2H]-furanone	4077-47-8	C_7_H_10_O_3_	142.2	1610	610.935	1.196
28	2-Ethylbutanoic acid	88-09-5	C_6_H_12_O_2_	116.2	1656	674.212	1.261
29	1-Butanoic acid	107-92-6	C_4_H_8_O_2_	88.1	1678	705.562	1.173
30	1-Methyl-2-pyrrolidinone	872-50-4	C_5_H_9_NO	99.1	1679	708.061	1.433
31	3-Methyl butanoic acid	503-74-2	C_5_H_10_O_2_	102.1	1731	791.146	1.230
32	1	*	*	*	1715	763.843	1.548
33	2	*	*	*	1393	383.229	1.148
34	3	*	*	*	1393	383.483	1.509
35	3-Methyl-2-butanol	598-75-4	C_5_H_12_O	88.1	1135	220.306	1.231
36	4	*	*	*	1201	254.184	1.083
37	(2,6)-Dimethylpyrazine	108-50-9	C_6_H_8_N_2_	108.1	1322	329.202	1.135
38	5	*	*	*	1311	321.404	1.066
39	6	*	*	*	1281	301.238	1.210
40	7	*	*	*	1278	299.894	1.512
41	Ethyl-2-hydroxypropanoate	97-64-3	C_5_H_10_O_3_	118.1	1299	313.069	1.143
42	8	*	*	*	1310	320.598	1.278
43	(Z)-3-Hexen-1-ol	928-96-1	C_6_H_12_O	100.2	1366	361.467	1.229
44	3-Methyl-2-butenal-D	107-86-8	C_5_H_8_O	84.1	1156	230.749	1.361
45	3-Methyl-2-butenal-M	107-86-8	C_5_H_8_O	84.1	1158	231.692	1.093
46	2,3-Pentadione	600-14-6	C_5_H_8_O_2_	100.1	1077	194.686	1.218
47	9	*	*	*	731	92.735	1.275
48	10	*	*	*	1056	186.163	1.369
49	Dibutylamine	111-92-2	C_8_H_19_N	129.2	1066	190.301	1.276
50	(E)-2-Methyl-2-pentenal	14250-96-5	C_6_H_10_O	98.1	1166	235.924	1.165
51	S-Methyl propanethioate	5925-75-7	C_4_H_8_OS	104.2	1143	224.117	1.126
52	11	*	*	*	1145	225.366	1.340
53	2-Hexenal	505-57-7	C_6_H_10_O	98.1	1202	254.495	1.172
54	12	*	*	*	1243	277.856	1.459
55	3-Methyl-3-buten-1-ol	763-32-6	C_5_H_10_O	86.1	1258	287.057	1.164
56	Methyl propyl disulfide	2179-60-4	C_4_H_10_S_2_	122.2	1248	280.923	1.142
57	Methyl allyl disulfide	2179-58-0	C_4_H_8_S_2_	120.2	1258	287.227	1.108
58	3-Hexanone	589-38-8	C_6_H_12_O	100.2	1074	193.369	1.295
59	Dipropyl sulfide	111-47-7	C_6_H_14_S	118.2	1093	201.731	1.158

VOCs, Volatile organic compounds; CAS #, Chemical Abstracts Service Registry Number; MW, Molecular weight; RI, Retention index; Rt, Retention time; Dt, Drift time; D, Dimer; M, Monomer; and *unidentified.

### 3.3 Quantitative analysis of VOCs on the training cohort

**Figure 3:**
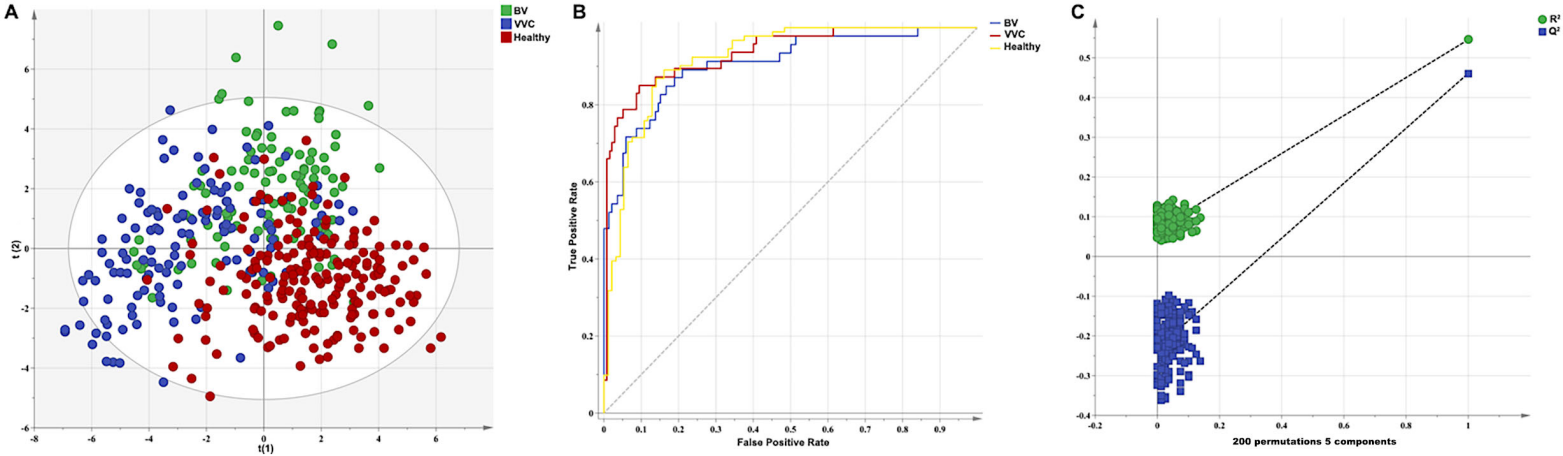
Performance evaluation of the PLS-DA model using a comprehensive dataset of VOCs. **(A)** PLS-DA score plot generated using a comprehensive dataset of VOCs from the different groups. **(B)** receiver operating characteristic curve for evaluating classification performance. **(C)** model validation with a permutation test (200 iterations).

A model was built using spectra of 70% of the samples (n = 106 BV, n = 127 VVC, and n = 199 healthy) and further cross-validated by PLS-DA. The initial variables were normalized by unit variance scaling. As shown in **Figure 3A**, the PLS-DA score plot showed a distinct separation among the three groups in two-dimensional space. The model parameters (R^2^X = 0.414, R^2^Y = 0.573, and Q^2^ = 0.511) suggested a robust predictive ability and acceptable goodness of fit. Additionally, a receiver operating characteristic curve analysis produced area under the curve (AUC) values of 0.91, 0.93, and 0.92 for the BV, VVC, and healthy groups, respectively (**Figure 3B**), demonstrating the model’s exceptional reliability. A permutation test (200 iterations) was conducted, which yielded R^2^ and Q^2^ intercepts of 0.071 and −0.223, respectively, indicating that the PLS-DA model was not overfitted (**Figure 3C**). Collectively, these results confirmed the robustness and reliability of the established model.

The model was subsequently assessed by seven-fold cross-validation. The confusion matrix (**Table 2**) indicated an overall predictive accuracy of 84.26%. The sensitivities of the BV, VVC, and healthy control groups were 0.75, 0.83, and 0.90, respectively, and the specificities were 0.96, 0.94, and 0.85, respectively.

**Table 2:** Confusion matrix of the model cross-validation results on the training cohort.

Actual group	No. (n)	Predicted group (n)			Correct (%)
		BV	VVC	Healthy	
BV	106	80	7	19	75.47
VVC	127	5	105	17	82.68
Healthy	199	9	11	179	89.95
Total	432	94	123	215	84.26

BV, Bacterial vaginosis; VVC, Vulvovaginal candidiasis.

### 3.4 Diagnostic performance of VOCs in the test cohort

The remaining 30% of the dataset (n = 46 BV, n = 47 VVC, and n = 91 healthy) was used as an independent test set to assess the model’s predictive efficacy and generalization capability. As shown in **Table 3**, the confusion matrix indicated an overall predictive accuracy of 80.98%. The sensitivities of the BV, VVC, and healthy control groups were 0.67, 0.74, and 0.91, respectively, and the specificities were 0.95, 0.96, and 0.75, respectively, which aligned with the cross-validation results. Despite the test cohort achieving a slightly superior prediction accuracy for the healthy group (91.21%) compared to the training cohort (89.95%), this negligible difference statistically corresponds to a mere single sample variance (1/91). These findings suggested a superior generalization capability of the model and robust predictive accuracy for novel vaginal samples, indicating the potential use of the model to diagnose BV and VVC.

**Table 3:** Confusion matrix of the model cross-validation results on the test cohort.

Actual group	No. (n)	Predicted group (n)			Correct (%)
		BV	VVC	Healthy	
BV	46	31	3	12	67.39
VVC	47	1	35	11	74.47
Healthy	91	6	2	83	91.21
Total	184	38	40	106	80.98

BV, Bacterial vaginosis; VVC, Vulvovaginal candidiasis.

### 3.5 Screening of potential VOC biomarkers

To further clarify the distinct metabolic characteristics influencing group differentiation, we assessed the effect of each variable using VIP scores and *P*-values. As shown in **Table 4**, nine important chemicals were identified as substantial contributors to the model based on the stringent criterion of VIP > 1 and *P* < 0.05. However, given that toluene is not a typical microbial metabolite, it was excluded from the subsequent PLS-DA modeling, and the model was further refined using the remaining eight compounds, which demonstrated strong potential as diagnostic biomarkers for differentiating BV and VVC from healthy controls.

**Table 4:** Potential indicators screened by the PLS-DA model.

No.	VOCs	RI	Dt (a.u.)	VIP Score	*P-*value
1	Propanoic acid-M	1557	1.112	1.1939	0.0000
2	Propanoic acid-D	1558	1.269	2.0948	0.0080
3	3-Methyl-2-butenal-M	1156	1.361	1.7102	0.0000
4	3-Methyl-2-butenal-D	1158	1.093	1.5418	0.0000
5	2,3-Pentadione	1077	1.218	1.4082	0.0000
6	2-Butanone, 3-hydroxy-M	1240	1.334	1.2565	0.0000
7	2-Butanone, 3-hydroxy-D	1238	1.070	1.2197	0.0000
8	Toluene	1038	1.012	1.1523	0.0002
9	2-Methyl propanoic acid	1595	1.157	1.1425	0.0001

VOCs, Volatile organic compounds; D, Dimer; M, Monomer; and VIP, Variable importance in projection.

### 3.6 Diagnostic performance of the eight finalized biomarkers in the entire cohort

**Figure 4:**
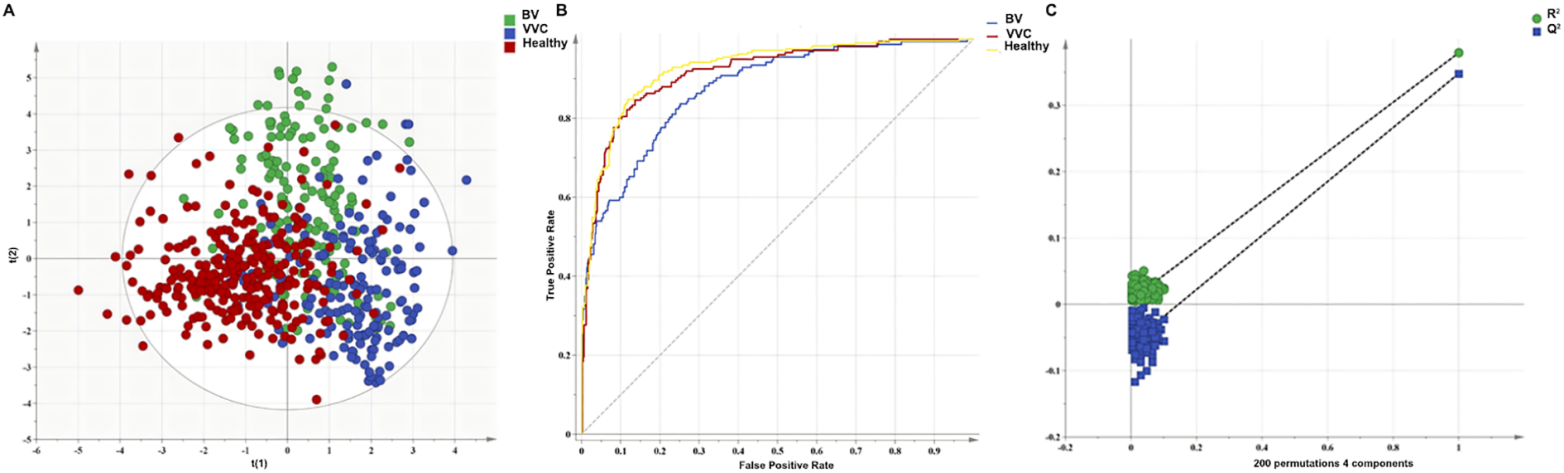
Performance evaluation of the PLS-DA model using eight biomarkers. **(A)** PLS-DA score plot generated using eight biomarkers from the different groups. **(B)** receiver operating characteristic curve to evaluate classification performance. **(C)** model validation with a permutation test (200 iterations).

To rigorously evaluate the clinical diagnostic value of the selected biomarkers, we developed an enhanced PLS-DA model employing the finalized panel of eight distinct microbial VOCs. This improved model demonstrated distinct spatial separation of the clinical cohorts (**Figure 4A**), resulting in strong goodness-of-fit and predictive metrics (R^2^X = 0.644, R^2^Y = 0.451, Q^2^ = 0.422). The diagnostic reliability was further validated by a receiver operating characteristic (ROC) analysis, which exhibited remarkable discriminatory power; the model produced AUC values of 0.88, 0.92, and 0.93 for the BV, VVC, and healthy groups, respectively (**Figure 4B**). A rigorous 200-iteration permutation test validated the model’s structural integrity and demonstrated the lack of overfitting, as indicated by markedly diminished intercepts (R^2^ = 0.0197, Q^2^ = -0.231) (**Figure 4C**). After conducting internal validation using seven-fold cross-validation, the multi-biomarker panel achieved an overall prediction accuracy of 78.25% for the entire cohort (**Table 5**). The model demonstrated remarkable specificity, particularly in effectively excluding false positives for BV (0.96) and VVC (0.87). The sensitivities for the BV, VVC, and healthy groups were 0.57, 0.82, and 0.88, respectively, with specificities of 0.96, 0.87, and 0.82, respectively. These findings collectively highlight the panel’s significant potential as a precise, non-invasive diagnostic instrument.

**Table 5:** Confusion matrix of the diagnostic performance using eight biomarkers.

Actual group	No. (n)	Predicted group (n)			Correct (%)
		BV	VVC	Healthy	
BV	152	86	31	35	56.58
VVC	174	7	142	25	81.61
Healthy	290	10	26	254	87.59
Total	616	103	199	314	78.25

BV, Bacterial vaginosis; VVC, Vulvovaginal candidiasis.

## 4 Discussion

The main aim of this study was to develop and validate a new VOC-based diagnostic method for BV and VVC using HS-GC-IMS technology. Our results indicated that the PLS-DA classifier developed in this study demonstrated enhanced diagnostic efficacy, with overall accuracies of 84.26% and 80.98% on the training and testing sets, respectively. The model demonstrated high classification performance, with AUC values greater than 0.90 across all cohorts. These findings are clinically important given the limitations of existing diagnostic criteria for BV and VVC. Conventional techniques, such as the Nugent score and wet mount analyses, are standard methods used in clinical laboratories (Workowski et al., 2021; Muzny et al., 2023). However, the Nugent score used to detect BV is labor-intensive and significantly reliant on operator skill, and wet mount microscopy used to detect VVC has a low sensitivity and is prone to false-negative results (Danby et al., 2021; Abou Chacra et al., 2024). Although the increasing use of nucleic acid amplification tests has improved sensitivity, their widespread use in basic healthcare has been limited by exorbitant costs and prolonged turnaround times (Coleman and Gaydos, 2018; Schwebke et al., 2020; Lev-Sagie et al., 2023). In these situations, HS-GC-IMS offers a viable diagnostic option. With a rapid detection time of 20 minutes, it efficiently improves upon both classical microscopy and molecular diagnostics. HS-GC-IMS provides the objectivity and sensitivity frequently lacking in microscopy, while preserving the high speed and cost-effectiveness usually sacrificed by molecular methods.

In the PLS-DA model, the monomer and dimer peaks of the same chemical produced different VIP ratings. This happens because the chemometric algorithm assesses each extracted ion signal as a mathematically independent variable within the data matrix, instead of determining the absolute total concentration of the substance. We explored the specific biochemical signatures supporting this diagnostic method by identifying nine critical VOCs using stringent selection criteria (VIP > 1 and *P* < 0.05). Propanoic acid, 3-methyl-2-butenal, and 3-hydroxy-2-butanone (all detectable as both monomers and dimers), along with 2,3-pentadione, and 2-methyl propanoic acid, were identified as potential contributors providing the molecular foundation for distinguishing BV and VVC patients from healthy individuals. Among these contributors, propanoic acid is a primary fermentation byproduct of numerous anaerobic bacteria (Mu et al., 2023; Pelayo et al., 2024). During the clinical progression of BV, anaerobic pathogens such as *Gardnerella vaginalis*, *Atopobium vaginae*, and *Prevotella* species proliferate excessively, potentially generating propanoic acid through mixed-acid fermentation pathways (Srinivasan et al., 2015; Ceccarani et al., 2019). Additionally, a newly recognized vaginal species, *Vaginimicrobium propionicum*, was confirmed to significantly generate propanoic acid (Diop et al., 2020). Its presence in the vaginal secretions of patients with BV was consistent with the increased propanoic acid levels noted in the VOC profile. Similar to propanoic acid, 2-methyl propanoic acid is another significant marker of anaerobic metabolism but has a different source. It is mainly generated by the breakdown of vaginal peptides and branched-chain amino acids, particularly valine (Liu et al., 2023a). BV-associated anaerobes, including *Prevotella* and *Porphyromonas* species, may promote this proteolytic process, leading to the substantial accumulation of 2-methyl propanoic acid in individuals with BV (Srinivasan et al., 2015; Dufresne, 2025).

Unlike BV, which is marked by the proliferation of anaerobic bacteria, VVC is predominantly caused by fungi, particularly *Candida albicans* (Bhosale et al., 2025; Liu et al., 2025). Consistent with this etiology, 3-hydroxy-2-butanone, 2,3-pentanedione, and 3-methyl-2-butenal were identified in this study as definitive markers of fungal energy metabolism and amino acid production. 3-Hydroxy-2-butanone is a prominent marker of fungal glucose fermentation (Li et al., 2014). In individuals with VVC, *Candida* can form biofilms, producing a hypoxic and acidic milieu that directly promotes 3-hydroxy-2-butanone production (Rodríguez-Cerdeira et al., 2020). At the same time, fungal overgrowth enhances amino acid metabolism, including valine and isoleucine synthesis that can cause 2,3-pentadione to accumulate (Krogerus and Gibson, 2013). Here, the identification of 3-methyl-2-butenal, an unsaturated aldehyde produced by the degradation of leucine and isoleucine or isoprenoid pathways, was consistent with earlier research that recognized this compound as a persistent extracellular metabolite of *Candida* species (Schuster et al., 2012; Costa et al., 2020).

Interestingly, toluene was identified as an indicators. It is not specifically a microbial metabolite, and therefore, its differences among the groups were likely due to external exposure and matrix effects. Patients who suffer from uncomfortable vaginitis frequently increase their use of sanitary products; because these products may contain trace aromatic compounds, their use may potentially increase toluene levels relative to those of healthy controls (Germolus et al., 2025; Syamson et al., 2026). As toluene is not a typical microbial metabolite, it was omitted from the next PLS-DA modeling of the selected VOCs.

This study has certain limitations. Initially, the baseline clinical characteristics, including body mass index and parity, were not thoroughly documented for the entire cohort of 616 participants, and these demographic variables might affect the VOCs profile. Second, 12 detected VOCs remained unidentified. This limitation exists due to the absence of reference standards for numerous complex microbial metabolites in existing commercial GC-IMS databases, and the initial investigation did not use parallel GC-MS or GC×GC-MS analysis. While this limits mechanistic depth, their chemical families can be provisionally deduced from their retention indices on the polar MXT-WAX column (Von Mühlen and Marriott, 2011). The lowest-RI peak (731) likely signifies a highly volatile short-chain alkane; the majority (RIs 1056-1393) denote medium-chain alcohols, aldehydes, or esters; and the highest-RI peak (1715) implies a strongly polar volatile fatty acid or a heavier heterocyclic complex (Arulvasan et al., 2025; Lu et al., 2022). Third, although the qualitative identification of the VOC biomarkers was meticulously cross-validated using the NIST RI database and an IMS Dt database derived from pure analytical standards, we did not independently co-inject pure chemical standards of the biomarker candidates with our clinical samples. Consequently, future research using pure analytical standards under uniform HS-GC-IMS settings are essential to conclusively validate these biomarker designations. Further, our research primarily focused on differentiating mono-infections (BV or VVC). In real-world clinical practice, co-infections of BV and VVC are frequently encountered (Xiao et al., 2022; Sobel and Vempati, 2024). We must recognize that the current PLS-DA algorithm, which is trained exclusively on pure metabolic profiles, may encounter difficulties in accurately classifying mixed infections. Although the present investigation established the fundamental baseline VOC signatures for these specific vaginitis types, it is essential to assess and enhance the diagnostic accuracy of HS-GC-IMS for coinfections. In the future, we may increase the sample size to specifically include mixed-infection cohorts and formulate classification algorithms that include mixed infections.

## 5 Conclusions

This study demonstrated that HS-GC-IMS is an expedient, non-invasive method for distinguishing between BV and VVC. The PLS-DA model demonstrated strong diagnostic efficacy, achieving predictive accuracies of 80% and AUC values exceeding 0.90 on both the training and testing cohorts. Eight VOC biomarkers were identified that produced unique metabolic fingerprints of anaerobic and fungal infections. Based on these biomarkers, the PLS-DA model had an overall prediction accuracy of 78.25% across the entire cohort of 616 participants.

## Data Availability

The raw data supporting the conclusions of this article will be made available by the authors, without undue reservation.
